# Fusion of Deep Convolution and Shallow Features to Recognize the Severity of Wheat *Fusarium* Head Blight

**DOI:** 10.3389/fpls.2020.599886

**Published:** 2021-01-21

**Authors:** Chunyan Gu, Daoyong Wang, Huihui Zhang, Jian Zhang, Dongyan Zhang, Dong Liang

**Affiliations:** ^1^Institute of Plant Protection and Agro-products Safety, Anhui Academy of Agricultural Sciences, Hefei, China; ^2^National Engineering Research Center for Agro-Ecological Big Data Analysis & Application, Anhui University, Hefei, China; ^3^Water Management and Systems Research Unit, USDA Agricultural Research Service, Fort Collins, CO, United States; ^4^Macro Agriculture Research Institute, College of Resource and Environment, Huazhong Agricultural University, Wuhan, China

**Keywords:** *Fusarium* head blight, transfer learning, Relief-F, fusion feature, random forest

## Abstract

A fast and nondestructive method for recognizing the severity of wheat *Fusarium* head blight (FHB) can effectively reduce fungicide use and associated costs in wheat production. This study proposed a feature fusion method based on deep convolution and shallow features derived from the high-resolution digital Red-green-blue (RGB) images of wheat FHB at different disease severity levels. To test the robustness of the proposed method, the RGB images were taken under different influence factors including light condition, camera shooting angle, image resolution, and crop growth period. All images were preprocessed to eliminate background noises to improve recognition accuracy. The AlexNet model parameters trained by the ImageNet 2012 dataset were transferred to the test dataset to extract the deep convolution feature of wheat FHB. Next, the color and texture features of wheat ears were extracted as shallow features. Then, the Relief-F algorithm was used to fuse the deep convolution feature and shallow features as the final FHB features. Finally, the random forest was used to classify and identify the features of different FHB severity levels. Results show that the recognition accuracy of the proposed fusion feature model was higher than those of models using other features in all conditions. The highest recognition accuracy of severity levels was obtained when images were taken under indoor conditions, with high resolution (12 MB pixels), at 90° shooting angle during the crop filling period. The Relief-F algorithm assigned different weights to the features under different influence factors; it made the fused feature model more robust and improved the ability to recognize wheat FHB severity levels using RGB images.

## Introduction

*Fusarium* head blight (FHB) mainly caused by *Fusarium graminearum* is a devastating disease of wheat and has a serious impact on wheat production worldwide, especially in China (Huang and Mcbeath, 2010). FHB-infected wheat will produce deoxynivalenol (DON) toxin that is poisonous to humans or animals and can persist for a long time in the food chain (Palacios et al., 2017; Peiris et al., 2017). If FHB can be detected effectively and disease severity level can be determined precisely, it can be controlled timely by applying fungicides. Particularly, the right number of doses of fungicides can be appropriately allocated according to the severity level to reduce the cost of fungicide application and protect ecological environment to a great extent (Yuan and Zhang, 2000). Recognition of wheat FHB was usually performed visually by experienced plant protectors in fields (Fernando et al., 2017). It is subjective, time-consuming, and laborious. Recently, some studies have utilized hyperspectral technology and image processing technologies for FHB recognition. Hyperspectral technology has high-level technical requirements and high costs. And also, it has a high demand for the natural environments such as light and wind and so on when collecting hyperspectral data (Bauriegel et al., 2011; Jin et al., 2018). Image processing technologies have strong generality, high efficiency, low cost, and low operating requirements in disease recognition (Mohd et al., 2019; Pantazi et al., 2019). Red-green-blue (RGB)–based images have been widely used in wheat crops. Although some valuable progress has been made (Jin et al., 2017; Aarju and Sumit, 2018), there is still a need to improve rating FHB severity level accurately by utilizing RGB images.

The first key request is to extract effective features from RGB images (Wang and Paliwal, 2003). Zahra and Davud (2015) extracted texture, color, and shape features of RGB images to recognize wheat fungal diseases with an accuracy of 98.3%. Frederic and Pierre (2007) extracted color and texture features of wheat ear RGB images to identify the wheat ear regions. Liu and Cui (2015) distinguished wheat from the background based on RGB and Lab color space and used the random forest (RF) algorithm to accurately segment the targeted winter wheat from RGB image of the canopy. The abovementioned color and texture features are widely used for crop disease identification (Zhu et al., 2017; Xiao et al., 2018), so they are fundamental references for wheat FHB identification. Among color features, the RGB channel, HSV channel, and Lab channel are widely used because they can effectively express the differences between diseased and other areas (Pydipati et al., 2006; Meunkaewjinda et al., 2008; Yao et al., 2009). Among texture features, the gray-level co-occurrence matrix (GLCM) can reflect the comprehensive information of the image about the direction, adjacent interval, and amplitude change (Chaki et al., 2015; Gavhale et al., 2015; Xie and He, 2016). Compared to color and texture features, deep convolution feature can well excavate the deep features information in images (Shervin et al., 2016; Lu et al., 2017). The LeNet-5 model proposed by Yann et al. (1998) has made the convolutional neural network achieve excellent results in the field of handwritten digit recognition for the first time and established the reputation of convolutional neural network in image recognition. In the ImageNet 2012 competition, the AlexNet deep convolutional neural network proposed by Alex et al. (2012) won the championship. Its classification results were much better than other traditional machine learning classification algorithms. The AlexNet deep convolutional neural network has attracted widespread attention since it was used in crop identification with good accuracy (Mostafa et al., 2017; Wei et al., 2018).

The accuracy of disease recognition from RGB images depends on the contribution from each extracted feature. Researchers have proposed feature selection algorithms for feature screening (Mitra et al., 2002; Marko and Igor, 2003; Valliammal and Geethalakshmi, 2012). Peng et al. (2005) proposed a minimal-redundancy-maximal-relevance criterion (mRMR) algorithm. The basic idea of mRMR was to use the theory of relevance in information theory and the size of mutual information as a measure of the correlation between features, as well as the sexual standards of features and category labels. Kira and Rendell (1992) proposed the Relief-F algorithm, which assigns different weights to all features according to the relevance of each feature and category. This algorithm is favorable by researchers because of its high efficiency, good results, and no limitation on data types (Durgabai et al., 2014; Wang et al., 2016).

Therefore, in this study we proposed the following procedure, especially a feature fusion method, to recognize the severity of wheat FHB. First, the deep convolution features of RGB images was extracted using the AlexNet convolutional neural network, and the color features and texture features of the images were extracted as shallow features. Next, to improve the recognition accuracy of wheat FHB severity levels, the Relief-F algorithm was used to fuse the extracted deep convolution feature and shallow features. Finally, the RF algorithm (Bosch et al., 2007) was used to model the features under different influence factors to explore the performance of the fusion features.

## Materials and Methods

### Study Area and Image Acquisition

**FIGURE 1:**
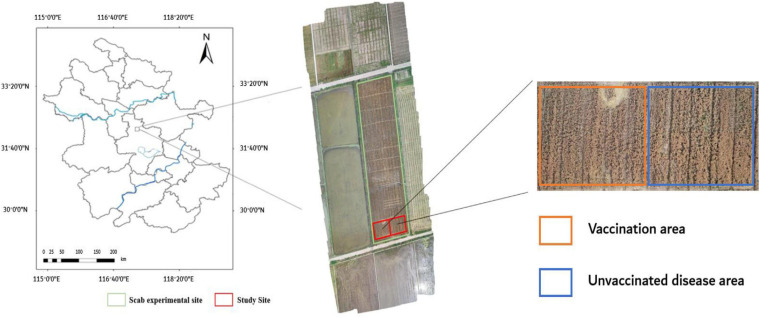
Location of the study site and an RGB image of the experimental plot was taken by a DIJ Spirit 4Pro drone at an altitude of 50 m on May 14, 2018.

The experimental base in this study locates at Anhui Academy of Agricultural Sciences (117°14′E, 31°53′N), in Auhui Province, China. The field experiments of wheat FHB were conducted from April 28, 2018 (flowering period), to May 14, 2018 (ripening period). Figure 1 shows the experimental field, which was divided into two sections: one was inoculated with FHB fungus inoculation, and the other was naturally grown. The inoculation section was gradually infected to form different levels of infection. A Nikon D3200 camera (Table 1) (effective pixels 6,016 × 4,000, focal length: 26 mm, aperture: f/8, exposure time: 1/250 s) was used to collect wheat ear images on sunny and cloudless days to reduce image distortion due to changing weather conditions. A total of 3,600 images of the wheat ear with FHB infection were collected. Among them, 1,200 images were randomly selected for the AlexNet learning, and the remaining 2,400 images were used for wheat FHB classification.

**TABLE 1:** Nikon D3200 manufacturing parameters.

**Nikon D3200 manufacturing parameters**	
Boundary dimension	125 × 96 × 76.5 mm
Sensor type	CMOS
Sensor size	APS frame (23.2 × 15.4 mm)
Maximum pixels	24.72 million
Effective pixels	24.16 million
Image processor	EXPEED 3
Image resolution	16 × 4,000 (L), 4,512 × 3,000 (M), 3,008 × 2,000 (S)

Images were acquired to ensure that only one wheat ear was in the lens with a black cloth as background, while the following influence factors were considered (Table 2):

**TABLE 2:** RGB images of the wheat ears were taken under different influence factors.

**Influence factor**			**Control influence factors**			
	
			**Light**	**Camera shooting angle (degrees)**	**Image resolution (MB pixels)**	**Wheat growth period**
Design of influence factors in the experiment	Light	Indoor		90	12	Filling
		Outdoor		90	12	Filling
	Shooting angle (degree)	30	Outdoor		12	Filling
		45	Outdoor		12	Filling
		90	Outdoor		12	Filling
	Image resolution (MB pixels)	12	Indoor	90		Filling
		6	Indoor	90		Filling
	Wheat growth period	Flowering	Outdoor	90	12	
		Filling	Outdoor	90	12	
		Ripening	Outdoor	90	12

1. Light condition
  Outdoor—under the influence of natural light and the light is uneven.
  Indoor—to minimize the influence of other illumination, a halogen lamp provides the light source in the dark room to make the wheat ears receive the light evenly.
2. Shooting angle
  The angle of camera lens and wheat ear was set up at 30°, 45°, and 90° (Zhao et al., 2013).
3. Image resolution
  Nikon D3200 was adjusted to the resolution of 12 million effective pixels (4,512 × 3,000) and 6 million effective pixels (3,008 × 2,000), respectively.
4. Wheat growth period
  Flowering, filling, and ripening period. The data were collected in the middle of each growth period.

**FIGURE 2:**
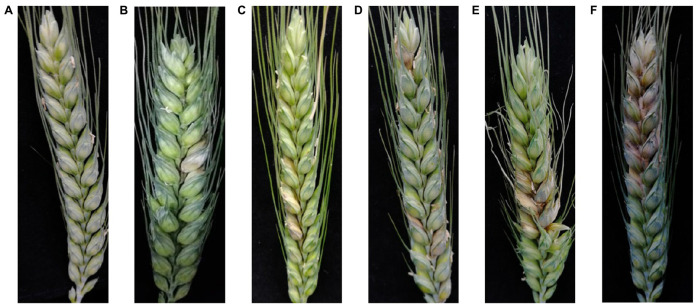
The images of FHB-infected wheat ears. **(A)** Healthy wheat ear with a disease level of 0; **(B)** wheat ear with a disease severity level of 1, **(C)** 2, **(D)** 3, **(E)** 4, and **(F)** 5.

While images were taken, the actual disease level of the ear in each image was manually identified by professional personnel. The GBT 15796-2011 Rules for Monitoring and Forecast of the Wheat Head Blight was referred to determine the infected level of FHB. The disease was classified into six levels based on the ratio (*R*) of wheat ear lesion area to wheat ear area, as Level 0: 0 ≤ *R* ≤ 0.01, Level 1: 0.01 < *R* ≤ 0.1, Level 2: 0.1 < *R* ≤ 0.2, Level 3: 0.2 < *R* ≤ 0.3, Level 4: 0.3 < *R* ≤ 0.4, and Level 5: *R* > 0.4 (Figure 2).

### Methods

**FIGURE 3:**
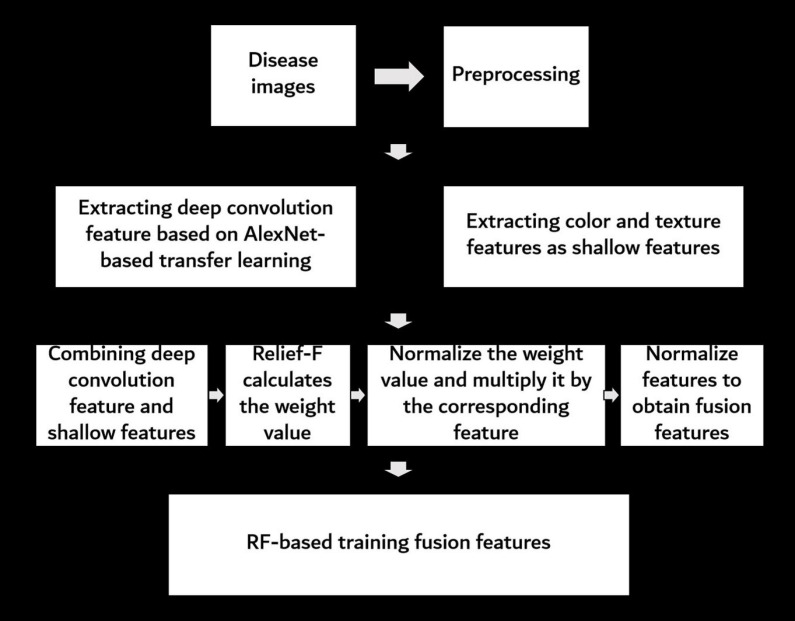
Flowchart of image processing procedure used in the study.

The overall procedure to determine the FHB-infected level by RGB images is shown in Figure 3. First, the raw images were preprocessed to remove interference information. Then, the deep convolution feature of the preprocessed images was extracted based on the AlexNet transfer learning, and then the color and texture features of the preprocessed images were extracted as shallow features. Next, the deep convolution feature and shallow features were merged, and the Relief-F algorithm was used to calculate the weights of the merged features. The weight values were normalized to make the weights more numerically comparable. Then, the weight value was multiplied by its corresponding feature. To improve the accuracy of the model, the final features were normalized and used as fusion features. Finally, all fusion features were input into a RF model to recognize the FHB severity level. Details on each step are given in the following sections.

#### Image Preprocessing

**FIGURE 4:**
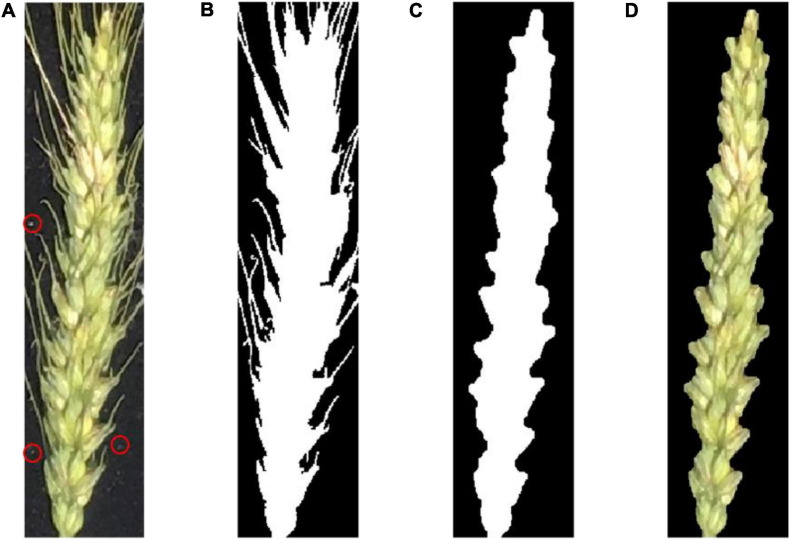
**(A)** A raw image of a wheat ear. The red circle indicates some fine dust on the black cloth; **(B)** binary image after removing background noise; **(C)** binary image after removing wheat awn from the ear; **(D)** pseudocolor image of the wheat ear in the area of interest.

Figure 4 shows an example of preprocessing raw images. First, the raw image was gray-scaled (Dougherty and Lotufo, 2003a), and the Otsu (Feige, 1999) threshold method was used for binarization. Next, a morphological region threshold filter (Dougherty and Lotufo, 2003b) was used to remove the noises such as the small dust on the black cloth. Then, a morphological open–close operation was used to remove the awn from the wheat ear to obtain the binary image of interest. Finally, the binary image was combined with the original image to produce a pseudocolor image.

#### Transfer Learning Based on the AlexNet to Extract Deep Convolution Feature

**FIGURE 5:**
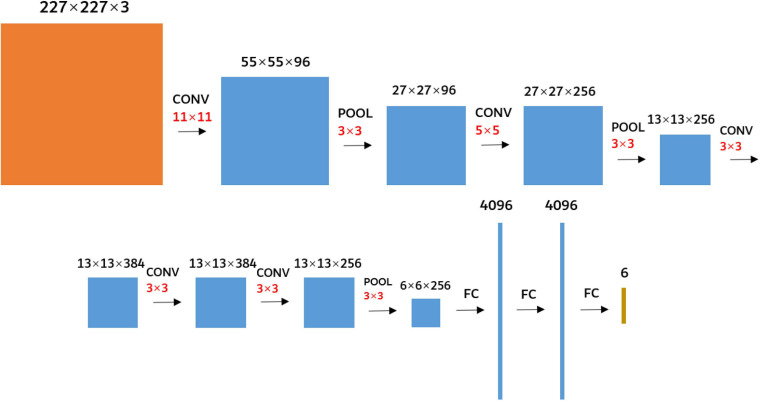
Diagram of the AlexNet network structure. The black numbers represent the size of the feature map. The red number represents the size of the kernels. CONV represents the convolution. POOL represents the maximum pooling. FC represents the fully connected.

The AlexNet (Alex et al., 2012) was used as the research network to extract the deep convolution feature of FHB images. It was not enough to train an excellent network with a small sample size, so the AlexNet model parameters trained using the ImageNet 2012 (Alex et al., 2012) dataset were transferred to FHB image sets for training. The parameters of the first five convolutional layers and corresponding pooling layers were retained, and the parameters of the three fully connected layers were trained. In addition, as the requested input of the AlexNet network was a 227 × 227 RGB image, the edges of the original FHB images were filled with 0 so that the aspect ratio of each image was 1. And then the images were resampled to 227 × 227 using bilinear interpolation (Kirkland, 2010). The network structure was shown in Figure 5.

A total of 1,200 images were used for the AlexNet transfer learning, where the training set had 840 images and the validation set had 360 images. The training parameters were set as follows: the learning rate was set to 0.0001, Maxepochs was set to 300, and the batch size was set to 20. The learning rate determines how quickly the parameter moves to the optimal value. Maxepochs represent the total number of trainings. The batch size indicates the number of samples used in each training batch in the training set. The final training time was 0.33 h, and the verification accuracy was 0.867. Results show that the AlexNet’s transfer learning could be used to recognize FHB, but accuracy was not good enough. Therefore, this study proposed to extract the deep convolution feature of the disease images through the network obtained by transfer learning and recognize the severity of the disease based on the deep convolution feature.

#### Shallow Features

The shallow features were extracted from the color and texture of FHB images as follows:

1. Color features: Select the B component of the RGB color space, the *a* component of the Lab color space (Gauch and Hsia, 1992), and the S component of the HSV color space (Sural et al., 2002) of the disease image to describe the color features. Among them, the *a* color ranges from dark green (low brightness value), gray (medium brightness value), to bright pink (high brightness value). S stands for saturation. The higher the saturation, the darker the color.

2. Texture features: The mean and variance of energy, entropy, inverse different moment, correlation, and contrast in GLCM (Haralick et al., 1973) were selected to describe the texture features of FHB images. Energy reflects the uniformity and texture of the gray distribution of the image. Entropy is a measure of the amount of information in the image. Inverse different moment reflects local changes in the texture of the image. Correlation reflects the consistency of the image texture. Contrast reflects the image sharpness and depth of texture grooves.

#### Feature Fusion of Deep Convolution Feature and Shallow Features

The deep convolution feature and shallow features were combined. The Relief-F algorithm was used to iterate 100 times to calculate the average weight of the combined features and normalize the weight value. The weight value was multiplied with its corresponding feature to obtain the weighted feature. Finally, the fused features were obtained by normalizing the weighted features to improve the accuracy of the model. The fusion formula was as follows:

(1) $$F\,{\text{weight}}_{l}=\frac{w_{l}}{\sum_{l=1}^{k}w_{l}}\times F\,{\text{cascade}}_{l}$$

(2) $$F\,{\text{normalization}}_{i,j}=\frac{F\,{\text{weight}}_{i,j}}{\sum_{j=1}^{n}F\,{\text{weight}}_{i,j}}$$

where _*Fcascade_l*_ is the *l* feature set specially collected for cascade sample, *l* = 1, 2,…, *k*, *k* is the feature dimension, _*w_l*_ is the feature weight of the *l*, _*Fweight_l*_ is the *l* feature set after calculating the weight; _*Fweight_i,j*_ is the eigenvalue of the row *i*, column *j* of the feature set after weight calculation, *i* = 1,2,…, *m*, *j* = 1,2,…, *n*, and *m* are the number of rows in the feature set, *n* is the number of columns in the feature set, and _*Fnormalization_i,j*_ is the normalized feature set.

**FIGURE 6:**
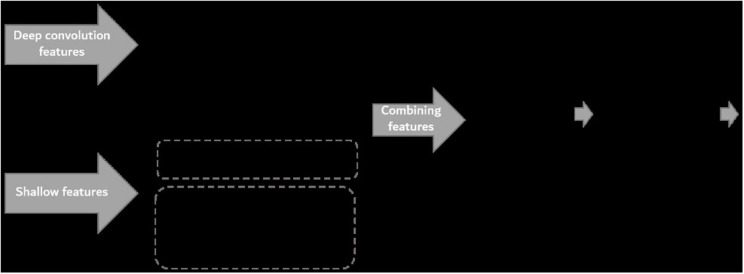
Structure of feature fusion network.

The feature fusion structure is given in Figure 6. The six-dimension deep convolution feature and shallow features (13-dimension) were extracted from input images. Relief-F was used to calculate the weights of the deep convolution feature (six-dimension) and shallow features (13-dimension) and the final fused features had 19 dimensions.

#### Random Forest

Random forest algorithm (Cutler et al., 2011) was a machine learning algorithm composed of multiple decision tree classification models. First, *N* training sets were extracted from the original dataset using the bootstrap (Cutler et al., 2011) sampling technique. Then, a classification regression tree was established for each training set, *N* decision tree models were generated, and *N* classification results were obtained. Finally, the prediction results of *N* decision trees were set to determine the category of the new sample by voting. RF gives results based on the prediction results of multiple decision trees, so even if some decision trees are misclassified, the final classification results were still correct.

## Results

The results were compared based on the measured severity level and the fusion feature classification. The algorithm was developed in Pycharm2017 and completed on Windows 10 PC with 12-core Intel core i7-6800k CPU (3.40 GHz), 16 GB RAM, and dual GTX1080Ti GPU. The influence factors of light, shooting angle, image resolution, and growth period were considered.

### Feature Extraction

**FIGURE 7:**
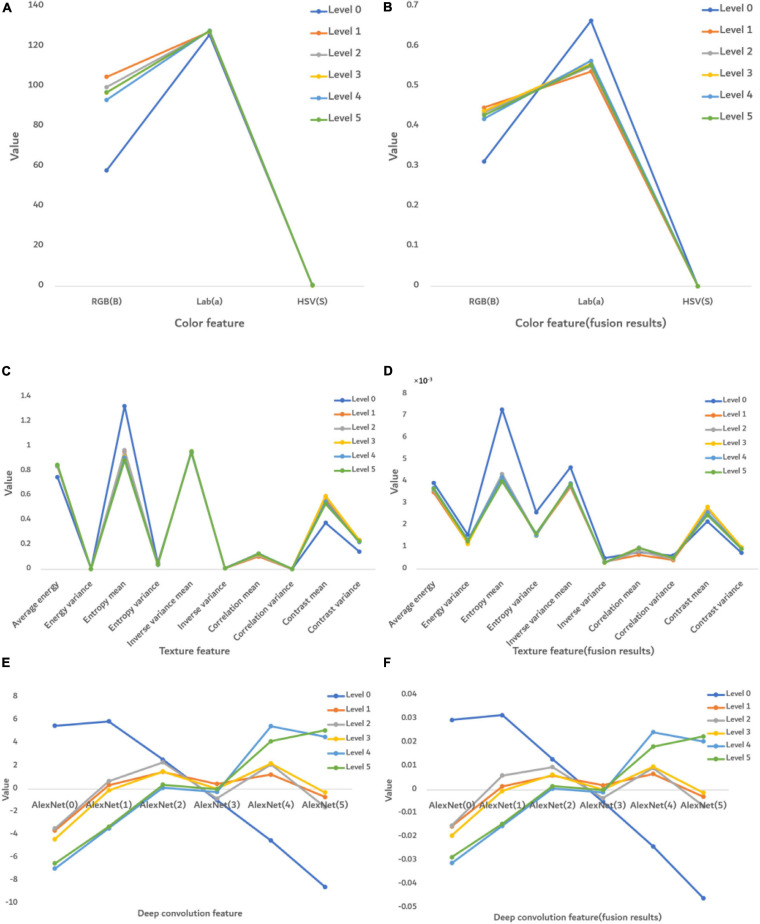
The numerical performance of features at each disease severity level. **(A)** Color features, **(B)** color features after fusion, **(C)** texture features, **(D)** texture features after feature fusion, **(E)** deep convolution feature, and **(F)** deep convolution feature after feature fusion. The color lines are the average eigenvalue of all samples at each severity level.

Figure 7 shows the numerical performance of features at each severity level. The value of each feature was obtained from averaging 2,400 images.

Before fusion (Figure 7A), only the RGB (B) feature was significantly different at each severity level, whereas after fusion (Figure 7B), the features were significantly different in RGB (B) and Lab (a). However, HSV(S) feature was the same before or after fusion, so this S feature is not able to distinguish FHB. The texture features before fusion could not effectively distinguish severity levels in Figure 7C, whereas after fusion they show some differences. It indicates that the ability of disease classification was improved by feature fusion. Figures 7E,F show that the deep convolution feature was able to distinguish disease severity levels both before and after fusion.

### Model Construction

To compare the performance of each model under different influence factors more intuitively, this study constructed each model based on the collected sample set and comprehensively evaluated the model results. The image distribution at each disease level under different influence factors was shown in Table 3.

**TABLE 3:** Number of images in the training and test set of each disease level under different influence factors.

**Influence factor**	**Sample set**	**Level 0**	**Level 1**	**Level 2**	**Level 3**	**Level 4**	**Level 5**
Light	Training set	90	307	164	157	117	215
Indoor	Test set	39	131	70	68	51	91
Outdoor	Test set	39	131	70	68	51	91
Angle	Training set	73	222	96	93	57	82
30°	Test set	32	96	42	42	27	38
45°	Test set	32	96	42	42	27	38
90°	Test set	32	96	42	42	27	38
Resolution	Training set	67	277	137	132	79	148
Low resolution	Test set	29	119	58	57	35	62
High resolution	Test set	29	119	58	57	35	62
Growth period	Training set	57	185	93	77	73	142
Flowering period	Test set	24	80	39	34	32	64
Filling period	Test set	24	80	39	34	32	64
Ripening period	Test set	24	80	39	34	32	64

**FIGURE 8:**
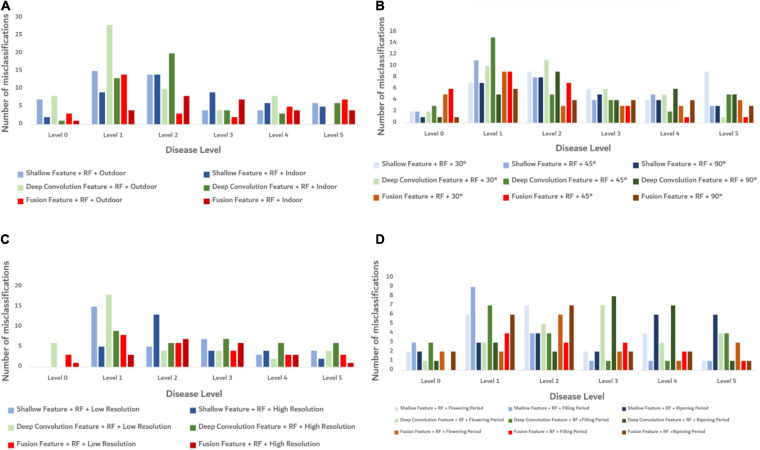
Classification error diagram of the models at each severity level under different conditions. **(A)** The misclassification of each model under different light conditions. **(B)** The misclassification of each model under different shooting angle conditions. **(C)** The misclassification of each model under different resolution conditions. **(D)** The misclassification of each model under different growth periods.

Random forest algorithm was used to build the models under different influence factors, and the classification errors of the models at each severity level are shown in Figure 8. Under all influencing factors, the fusion features model has the least number of misclassifications. When the disease levels are 1 and 2, each model has more misclassifications than other disease levels. It shows that the fusion features performed better than other features in the models under different influence factors with a smaller number of misclassifications. But when the disease level was 1 or 2, the recognition accuracy of each model is poor. To further study the efficiency and stability of the model, the 10-fold cross-validation (Kohavi, 1995) was used to cross-verify the data, and the predicted time of each model training was calculated. The results are shown in Table 4. The accuracy of each model was better under indoor than outdoor lighting conditions. Under three observation angles, the 90° observation angle was the best in terms of accuracy. The images with higher resolution appropriately improved model accuracy. The identification accuracy was the highest for images taken during the crop filling period. Considering both model accuracy and training prediction time, the fusion features proposed in this study performed better than using the shallow features or deep convolution feature independently under different influence factors. This result indicates that the fusion features had high accuracy and strong robustness in the recognition of FHB severity level.

**TABLE 4:** Model performance under different influence factors.

**Influence factor**		**Model**	**Model accuracy**	**Cross validation accuracy**	**Time (s)**
Light	Outdoor	Shallow features + RF	0.8889	0.8880	15.70
	Outdoor	Deep convolution feature + RF	0.8711	0.8746	13.34
	Outdoor	Fusion features + RF	0.9244	0.9213	16.25
	Indoor	Shallow features + RF	0.9000	0.9000	15.54
	Indoor	Deep convolution feature + RF	0.8956	0.8933	12.91
	Indoor	Fusion features + RF	0.9378	0.9310	15.87
Angle	30°	Shallow features + RF	0.8664	0.8685	7.02
	30°	Deep convolution feature + RF	0.8736	0.8783	6.41
	30°	Fusion features + RF	0.9025	0.9087	7.08
	45°	Shallow features + RF	0.8809	0.8804	6.92
	45°	Deep convolution feature + RF	0.8773	0.8728	6.35
	45°	Fusion features + RF	0.9025	0.9098	6.99
	90°	Shallow features + RF	0.8989	0.8925	6.99
	90°	Deep convolution feature + RF	0.8917	0.8807	6.90
	90°	Fusion features + RF	0.9206	0.9170	7.23
Resolution	Low resolution	Shallow features + RF	0.9056	0.9002	9.25
	Low resolution	Deep convolution feature + RF	0.8944	0.8920	7.67
	Low resolution	Fusion features + RF	0.9250	0.9225	9.16
	High resolution	Shallow features + RF	0.9222	0.9212	9.14
	High resolution	Deep convolution feature + RF	0.9056	0.9050	8.50
	High resolution	Fusion features + RF	0.9417	0.9339	9.33
Growth period	Flowering period	Shallow features + RF	0.9194	0.9165	6.98
	Flowering period	Deep convolution feature + RF	0.9158	0.9176	6.02
	Flowering period	Fusion features + RF	0.9414	0.9484	7.13
	Filling period	Shallow features + RF	0.9304	0.9286	6.95
	Filling period	Deep convolution feature + RF	0.9267	0.9264	6.26
	Filling period	Fusion features + RF	0.9524	0.9516	7.21
	Ripening period	Shallow features + RF	0.9158	0.9143	7.05
	Ripening period	Deep convolution feature + RF	0.9194	0.9165	6.31
	Ripening period	Fusion features + RF	0.9377	0.9407	6.92

## Discussion

### Analysis of Image Preprocessing Results

**FIGURE 9:**
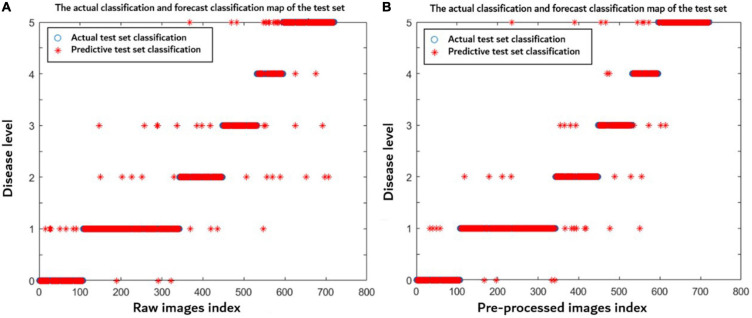
Disease level prediction distribution of each model using **(A)** raw images and **(B)** preprocessed images. The blue symbol indicates the actual disease severity level, and the red symbol indicates the predicted disease severity level.

In order to better discuss the advantages of preprocessing in this article, a total of 2,400 disease images under different influence factors were made into one sample set including 1,680 images in the training set and 720 images in the test sets. The raw and preprocessed images were used to build the models through the method proposed in this article, respectively. Figure 9 indicates that the model established using preprocessed images has a better prediction distribution of the disease level and higher model accuracy (0.943) than using the raw images (0.922). After preprocessing, the noises and wheat ears were removed, so the accuracy of the model was improved.

### Analysis of Fusion Feature

**FIGURE 10:**
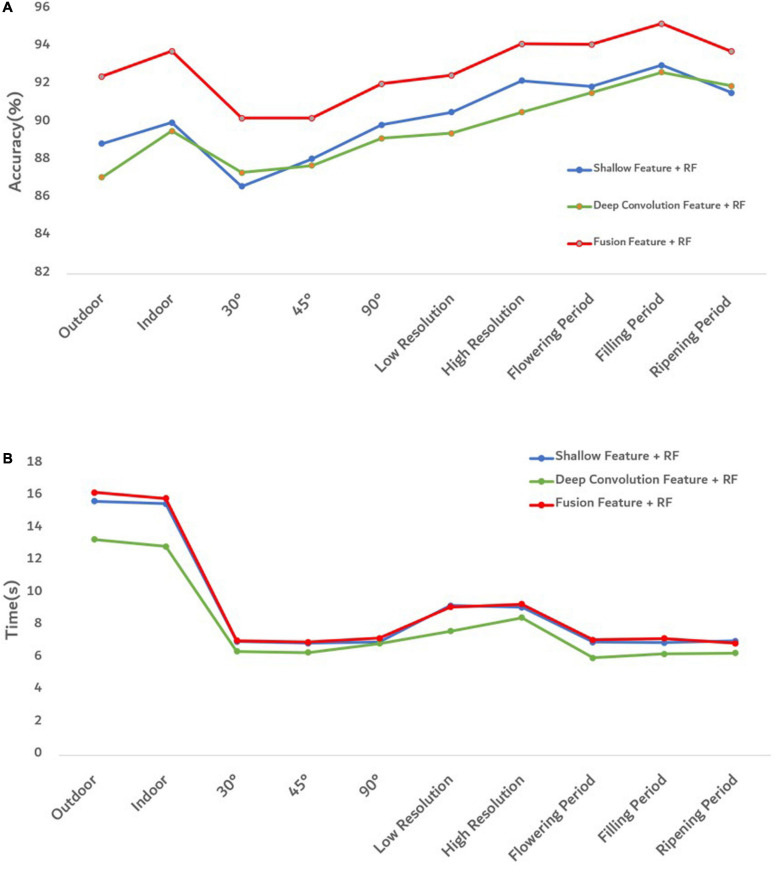
Evaluation diagram of each model under different influence factors. **(A)** The accuracy of each model. **(B)** The training and prediction time of each model.

To better evaluate the goodness of fusion features, we evaluated the goodness of fusion features through the accuracy of each model and the prediction time of model training (Figure 10).

**FIGURE 11:**
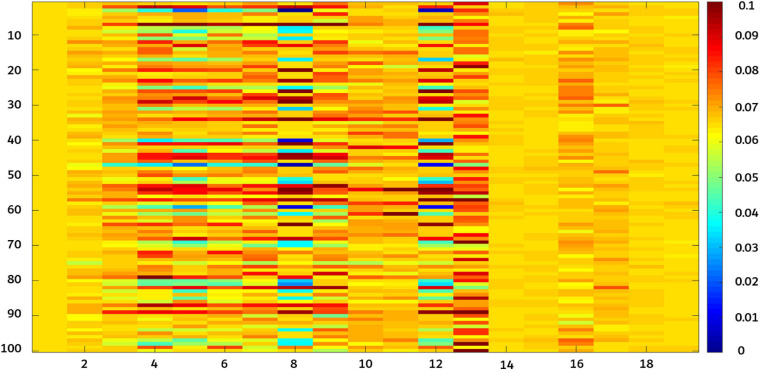
Relief-F iterates 100 times for each weight value. X-axis 1–6 correspond to deep convolution feature (AlexNet0, AlexNet1, AlexNet2, AlexNet3, AlexNet4, and AlexNet5); 7–9 correspond to color features [RGB(B), Lab(a), and HSV(S)]; and 10–19 correspond to texture features (average energy, energy variance, entropy mean, entropy variance, inverse different moment mean, inverse different moment variance, correlation mean, correlation variance, contrast mean, and contrast variance).

Figure 10A shows that the proposed fusion feature had a better performance on the accuracy of the model than the model constructed by other features. Under different influence factors, the proposed fusion method had stronger robustness, and model accuracy was greater than 90%, which was 2–5% higher than the recognition accuracy of deep convolution feature or shallow features. The efficiency of an algorithm is also important in practical applications (Yu et al., 2013). The predicted runtime of the model was used to evaluate the efficiency of the method. Figure 10B shows that although the fused features were higher in dimensions than other features, the model training time still performed good. To better analyze the effectiveness of the feature fusion method, FHB images with different influence factors were mixed as one sample set. After extracting the features of the sample set, 100 iterations were performed using Relief-F. The weight results obtained each time are shown in Figure 11. As the sample set was generated by mixing all influence factors, this increases the difficulty of training. In the meanwhile, the sample size of 2,400 was relatively small for deep learning. Therefore, the weight value of the deep convolution feature fluctuated greatly during the iteration process. For different growth periods and light conditions, the corresponding contribution of each color feature was different, resulting in a large fluctuation in the weight value of each color feature. For different resolution and shooting angles, the extracted texture features were different, so the weight values of texture features also fluctuated. Notice that the weight values of the deep convolution feature and shallow features fluctuated from low to high. It indicates that some features made more contributions when identifying certain influence factors than other factors. Therefore, when some features do not perform well in recognition, their weight values can be decreased so the weight values of other favorable features can be increased. Our results confirm that FHB image sets in this article can be well described by both deep convolution feature and the shallow features.

**FIGURE 12:**
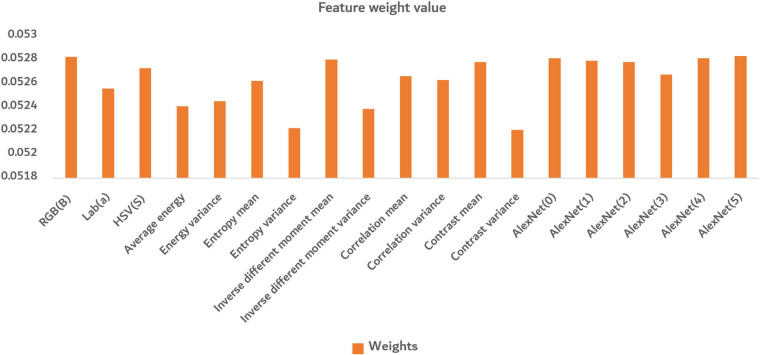
The average of final normalized weight value. The orange bar in the figure represents the weight value of different features calculated by the Relief-F algorithm.

In Figure 12, the bar in the figure represents the weight value of different features calculated by the Relief-F algorithm. The larger the value was, the greater the contribution of the feature made. There were similar maxima in both deep convolution feature and shallow features, indicating that both deep convolution feature and shallow features made great contributions to identifying disease severity levels. The shallow features can well reflect the situation of different disease levels from the color and texture information of the images. Among the color features, the channel a of Lab color space performed better. In the texture features, except for the entropy variance and contrast variance, the other features performed well. The deep convolution feature can discover deep information well through convolutional processing.

### Analysis of Different Influence Factors

**FIGURE 13:**
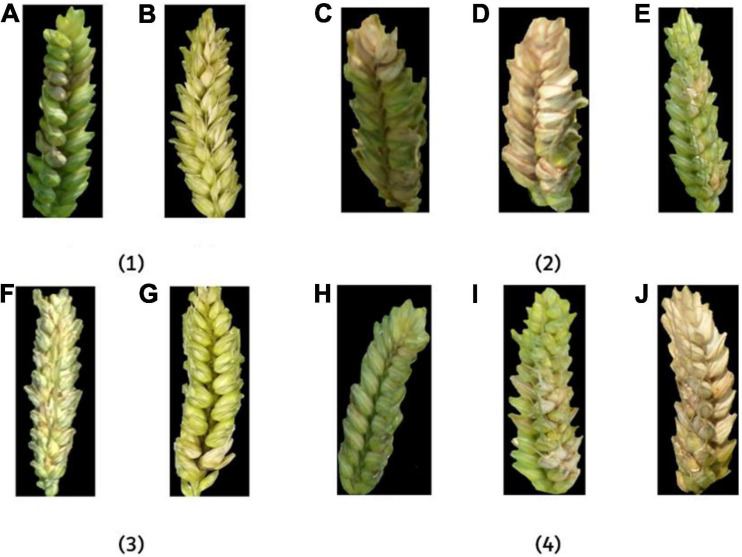
The example images of FHB-infected wheat ears under different influence factors. In (1), **(A)** outdoor and **(B)** indoor; in (2), **(C)** 30°, **(D)** 45°, and **(E)** 90°; in (3), **(F)** low-resolution and **(G)** high-resolution; in (4), **(H)** flowering period, **(I)** filling period, and **(J)** ripening period.

To investigate the influence of different factors on the fusion feature method, FHBs under different conditions were analyzed, respectively (Figure 13).

In Figure 13(1), the image taken in the outdoor environment was influenced by the difference of light and mirror effect (Barbedo, 2018), which has an impact on feature extraction. In an indoor environment, the image received relatively uniform light. Although there was a partial shadow in the gap between the ears in some images, it had little effect on disease recognition. However, it can be seen from the results that the accuracy difference of indoor and outdoor on the fusion feature model was less than 1% (Table 3), which shows that the fusion method has a certain resistance to the influence of light. In Figure 13(2), the larger the angle, the more information the camera can capture and the better the description of the disease. The smaller the angle, the more likely overlap will appear in the image and cause certain errors. According to the results, the recognition accuracy of 30° and 45° was similar, and the performance of 90° was the best among the three angles. In Figure 13(3), high-resolution images were more informative and performed well in feature extraction. It can be seen from the results that the accuracy of high-resolution images was higher than that of low-resolution images. In Figure 13(4), the color of the disease was not the same at different growth periods. FHB has just erupted during the flowering period, so the disease features were not obvious. FHB was more obvious in the filling period. The color difference between the normal wheat ear and FHB in the ripening period was small. Thus, the recognition accuracy during the filling period was the highest, and the recognition accuracy during the flowering period and the ripening period was basically the same.

To sum up, the model constructed with the fusion feature method has a certain resistance to different influence factors. More comparative experiments to explore a good collection environment should be considered in future research with valuable information provided by the study.

## Conclusion

The study proposed a method to recognize disease severity levels of FHB-infected wheat ears using RGB images, which were taken under different influence factors, such as light condition, shooting angle, image resolution, and crop growth period. The deep convolution feature and shallow features extracted from these images were analyzed as contrast experiments for FHB identification. The feature fusion method was then proposed based on the deep convolution and shallow features under different influence factors. Results show that the recognition accuracy of the fusion features model was higher than that of using the deep convolution feature or shallow features alone. The prediction time of the feature fusion model was good, and it performed more robust under different influence factors. The highest accuracy of recognizing severity levels was obtained when images were taken indoor, with high resolution (12 MB pixels), at 90° shooting angle and during the crop filling period. The proposed feature extraction method has significant advantages in the identification of wheat FHB disease severity levels and provides important technical support for plant protection in precision fungicide application and the development of disease control methods.

## Data Availability Statement

The raw data supporting the conclusions of this article will be made available by the authors, without undue reservation.

## Author Contributions

CG, DW, DZ, and DL: conceptualization. CG and DW: data curation. DW: formal analysis and methodology. DW and DZ: investigation and resources. DW, HZ, and DZ: writing – original draft. CG, HZ, and JZ: writing – review and editing. All authors contributed to the article and approved the submitted version.

## Conflict of Interest

The authors declare that the research was conducted in the absence of any commercial or financial relationships that could be construed as a potential conflict of interest.
